# Bioinformatics Analysis of Prognostic miRNA Signature and Potential Critical Genes in Colon Cancer

**DOI:** 10.3389/fgene.2020.00478

**Published:** 2020-06-09

**Authors:** Weigang Chen, Chang Gao, Yong Liu, Ying Wen, Xiaoling Hong, Zunnan Huang

**Affiliations:** ^1^Key Laboratory of Big Data Mining and Precision Drug Design of Guangdong Medical University, Research Platform Service Management Center, Guangdong Medical University, Dongguan, China; ^2^Key Laboratory for Research and Development of Natural Drugs of Guangdong Province, School of Pharmacy, Guangdong Medical University, Dongguan, China; ^3^The Second School of Clinical Medicine, Guangdong Medical University, Dongguan, China; ^4^Institute of Marine Biomedical Research, Guangdong Medical University, Zhanjiang, China

**Keywords:** colon cancer, microRNA, bioinformatics, prognosis, biomarker, TCGA, GEO

## Abstract

This study aims to lay a foundation for studying the regulation of microRNAs (miRNAs) in colon cancer by applying bioinformatics methods to identify miRNAs and their potential critical target genes associated with colon cancer and prognosis. Data of differentially expressed miRNAs (DEMs) and genes (DEGs) downloaded from two independent databases (TCGA and GEO) and analyzed by R software resulted in 472 DEMs and 565 DEGs in colon cancers, respectively. Next, we developed an 8-miRNA (hsa-mir-6854, hsa-mir-4437, hsa-mir-216a, hsa-mir-3677, hsa-mir-887, hsa-mir-4999, hsa-mir-34b, and hsa-mir-3189) prognostic signature for patients with colon cancer by Cox proportional hazards regression analysis. To predict the target genes of these miRNAs, we used TargetScan and miRDB. The intersection of DEGs with the target genes predicted for these eight miRNAs retrieved 112 consensus genes. GO and KEGG pathway enrichment analyses showed these 112 genes were mainly involved in protein binding, one-carbon metabolic process, nitrogen metabolism, proteoglycans in cancer, and chemokine signaling pathways. The protein–protein interaction network of the consensus genes, constructed using the STRING database and imported into Cytoscape, identified 14 critical genes in the pathogenesis of colon cancer (*CEP55*, *DTL*, *FANCI*, *HMMR*, *KIF15*, *MCM6*, *MKI67*, *NCAPG2*, *NEK2*, *RACGAP1*, *RRM2*, *TOP2A*, *UBE2C*, and *ZWILCH*). Finally, we verified the critical genes by weighted gene co-expression network analysis (WGCNA) of the GEO data, and further mined the core genes involved in colon cancer. In summary, this study identified an 8-miRNA model that can effectively predict the prognosis of colon cancer patients and 14 critical genes with vital roles in colon cancer carcinogenesis. Our findings contribute new ideas for elucidating the molecular mechanisms of colon cancer carcinogenesis and provide new therapeutic targets and biomarkers for future treatment and prognosis.

## Introduction

Colon cancer is one of the common malignant tumors of the digestive tract and occurs in the colon. With the development of the economy and the improvement of people’s living standards, the incidence of colon cancer in recent years has increased, and the age of onset lowered, posing a serious threat to people’s life and health (Arnold et al., 2017). Patients with colon cancer have no specific clinical symptoms in the early stage (Cappell, 2008). Most patients are in the middle and late stages when they seek medical treatment, and the treatment and prognosis are poor (Cappell, 2008). Most of the deaths of colon cancer patients are a result of tumor metastasis (Siegel et al., 2017). The 5-year survival rate of patients with metastatic colon cancer is much lower than that of non-metastatic colon cancer patients (Zhang et al., 2015). Therefore, it is necessary to identify new biomarkers and find potential therapeutic targets for early detection and treatment of colon cancer through effective strategies.

MicroRNAs (miRNAs) are short non-coding RNAs of approximately 18–25 nucleotides in length. Since their discovery, there has been a plethora of research indicating the aberrant expression of miRNAs in various types of cancers, including those of the colon, liver, and lung (Wang et al., 2015; Yang et al., 2015; Ding et al., 2018). MiRNAs can act as tumor suppressor genes or oncogenes in tumor tissues. Studies show that down-regulation of miR-708 expression could inhibit the progress of colon cancer cells by targeting the tumor promoter zinc finger E-box binding homeobox 1 (ZEB1), and overexpressed miR-155 could promote the proliferation of cancer cells by targeting the tumor suppressor cbl proto-oncogene (*CBL*) (Yu et al., 2017; Sun et al., 2019). Multiple high-throughput studies have shown high correlations between miRNA expression levels and the treatment and diagnosis of cancer patients (Bolmeson et al., 2011; Toiyama et al., 2014; Tan et al., 2018). In colon cancer, miRNAs are associated with the transmission and inhibition of numerous signaling pathways, and have great potential in diagnosis, prognosis, and personalized targeted therapy (Cekaite et al., 2016). It follows that in-depth studies of miRNAs will contribute to understanding the mechanism of colon cancer development and its biological functions, providing a theoretical basis for its prevention, diagnosis, and treatment.

Bioinformatics uses computational tools to store, search, and analyze biological information. A wide array of computational techniques related to database design and construction, protein structure and function prediction, gene discovery, and expression data clustering, are provided as bioinformatics methods for researching cancer and several other diseases (Luscombe et al., 2001). Access to The Cancer Genome Atlas (TCGA) (Tomczak et al., 2015), the Gene Expression Omnibus (GEO) (Barrett et al., 2007), the Kyoto Encyclopedia of Genes and Genomes (KEGG) (Kanehisa and Goto, 2000), the Gene Ontology (GO) database (Ashburner et al., 2000), and other databases are pertinent to cancer research. These resources enable relevant tumor data to be searched, processed, and analyzed by using differential expression analysis, survival analysis, functional enrichment analysis, pathway enrichment analysis, and the other functional tools available. Early biomarkers and potential therapeutic targets of tumors identified by these methods have assisted in exploring the molecular mechanisms of tumor pathogenesis and provide clues for further understanding of related tumors. For example, functional enrichment and survival analysis showed that miR-19b-3p might affect the apoptosis and proliferation of human colon cancer cells through SMAD family member 4 (SMAD4) and serve as a prognostic marker for colon cancer (Jiang et al., 2017). In another study, differentially expressed genes (DEGs) identified in colon cancer by differential expression analysis were further analyzed using function and survival analysis approaches (Yong et al., 2018). The results implicated protein phosphatase 2 catalytic subunit alpha (PPP2CA) in the occurrence and development of colon cancer, and its potential to serve as a therapeutic target in colon cancer (Yong et al., 2018). With the gradual development of molecular biology technology, bioinformatics has become increasingly important in cancer research, performing a major role in elucidating cancer mechanisms and finding novel targets for cancer treatment and patient prognosis.

Colon cancer is a multifactorial disease caused by assorted factors, such as genetic, environmental, and lifestyle influences, but its pathogenesis is not fully clarified (Aran et al., 2016). Exploring and studying the molecular mechanism and critical genes of colon cancer is key in improving the prevention and treatment of colon cancer. In this paper, we performed differential expression analysis to screen out miRNA (DEMs) and DEGs from colon cancer data downloaded from two independent databases (TCGA and GEO). To identify prognostic miRNAs, we constructed a Cox proportional hazards regression model. Then, we identified the overlapping genes between the predicted DEM targets and the DEGs and performed a functional enrichment analysis to understand the potential biological functions of these consensus genes. Finally, we constructed the protein–protein interaction (PPI) network of the consensus genes to illuminate the critical genes. These results might provide new ideas for future research and treatment of colon cancer by exploring prognostic miRNAs and therapeutic targets in colon cancer.

## Materials and Methods

### Tumor Data and Differential Expression Analysis

We downloaded 467 miRNA transcriptomes of 459 colon cancer and 8 normal tissue samples from the TCGA database on June 3, 2019, as well as the GSE24514 microarray data of 34 tumor tissues and 15 normal tissues from the GEO database. Both datasets were analyzed using R software (version 3.4.4) packages edgeR and limma, to identify DEMs and DEGs, respectively. The cutoff criteria were *P*_*adj*_ < 0.05 and |log_2_FC|> 1.0, where FC denotes fold change (Robinson et al., 2010; Ritchie et al., 2015).

### Cox Proportional Hazards Regression Model Based on DEMs

To evaluate the effect of single independent miRNAs on the survival time of colon cancer patients, we performed univariate Cox proportional hazard regression analysis (Ahmed et al., 2007) on DEMs using the survival package of R software and screened miRNAs related to patient survival according to the cutoff criterion of *P* < 0.01. Multivariate Cox proportional hazards regression analysis (Ahmed et al., 2007) with stepwise regression methods and a mathematical model allowed identifying prognostic miRNAs and evaluating the impact of these miRNAs on the survival distribution of patients. From the constructed Cox proportional hazards regression model, we used the following formula to compute the risk scores for each patient: miRNA risk score = β_*miRNA1*_ × exp(miRNA_1_) + β_*miRNA2*_ × exp(miRNA_2_) + … + β_*miRNAn*_ × exp(miRNA_*n*_), where β is the regression coefficient derived from the multivariate Cox proportional hazards regression model, and exp() is the expression level of prognostic miRNAs (Sui et al., 2017). This study divided the patients into a high-risk group and a low-risk group based on the median value of the risk score. The Kaplan–Meier survival curves of both groups were estimated. Then, we calculated the 5-year survival rates of the high-risk and low-risk groups and plotted the receiver operating characteristic (ROC) (Heagerty and Zheng, 2005) curve to test whether the predictive ability of the model was reliable.

### Target Genes Prediction for Prognostic miRNAs

To predict the target genes of the prognostic miRNAs, we used the online analysis tools TargetScan (Agarwal et al., 2015) and miRDB (Wong and Wang, 2015) on June 14, 2019. To further improve the reliability of these results, we identified the overlapping target genes by using the VennDiagram package of R software. Then, these overlapping target genes were crossed with DEGs by using the VennDiagram package of R software to obtain the consensus genes.

### Functional Enrichment Analysis of Consensus Genes

For GO and KEGG pathway enrichment analyses, we used the Database for Annotation, Visualization and Integrated Discovery (DAVID) (Huang da et al., 2009) and the KEGG Orthology-Based Annotation System (KOBAS) (Xie et al., 2011), respectively. *P* < 0.05 was set as the cutoff criterion.

### Construction and Analysis of PPI Networks With Consensus Genes

The Search Tool for the Retrieval of Interacting Genes (STRING) can aid in understanding the PPI by integrating a large number of known and predicted correlation data between proteins (Szklarczyk et al., 2017). To study the interactions between the consensus genes and to obtain potential critical genes, we constructed their PPI network using the STRING database on July 8, 2019. Genes with significant interactions were screened out based on a confidence score ≥0.4 (Sun et al., 2017), and the filtered results were imported into Cytoscape software (version 3.7.0) for network visualization (Shannon et al., 2003). We used the CentiScaPe plugin (Scardoni et al., 2014) for topology analysis of the entire network to calculate the central parameters, such as the degree value of each node in the PPI network (Williams and Del Genio, 2014). In consideration of the degree value of each node differing significantly, we calculated the average value of the degree of all nodes. Simultaneously, to obtain more meaningful target genes, we selected nodes with scores larger than twice the average as candidate hub nodes. Then, we used the Molecular Complex Detection (MCODE) plugin (Bader and Hogue, 2003) in Cytoscape to screen out the important functional modules in the PPI network of the consensus genes. The MCODE plugin parameters were degree cutoff ≥10, node score cutoff ≥0.2, *k*-core ≥2, and max depth = 100 (Zhao et al., 2018).

### Weighted Gene Co-expression Network Analysis

Weighted gene co-expression network analysis (WGCNA) allows analyzing the gene expression patterns of multiple samples for mining the core genes in the pathogenesis of patients with colon cancer (Langfelder and Horvath, 2008). This study analyzed 13,640 genes from the transcriptome data (GSE24514) using the WGCNA algorithm, and 49 samples were clustered through the systematic cluster tree to determine any outliers. Then, we set an appropriate soft threshold of 15 to make the co-expression network meet the scale-free distribution, and genes with similar expression patterns were merged into the same module using a dynamic tree-cutting algorithm (module size = 30) (Ning and Sun, 2020). Subsequently, three different-colored modules containing the most DEGs were further selected to mine the core genes. The edges with topological overlap measures greater than 0.30 were selected and input into Cytoscape for network visualization (Deng et al., 2018). Using the CentiScaPe plugin in Cytoscape, we calculated the degree value of each gene. Genes with degrees more than twice the average value were considered the core genes of the network.

### Running Scripts

All running scripts used above can be found in Supplementary Material.

## Results

### Differential Expression Analysis of Colon Cancer

**FIGURE 1:**
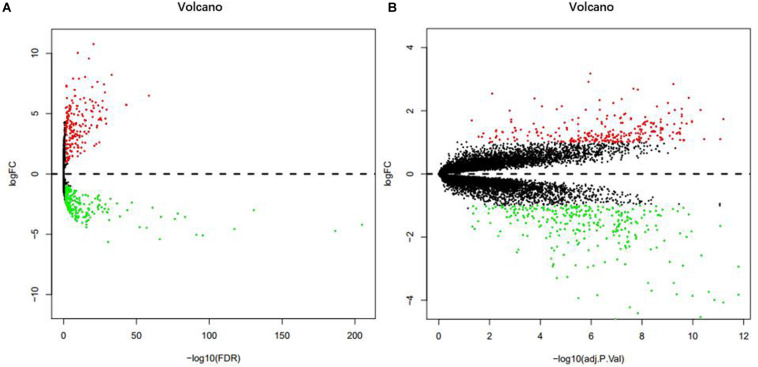
Volcano plot of DEMs in TCGA **(A)**. Volcano plot of DEGs in GSE24514 **(B)**. Red dots represent up-regulation and green dots represent down-regulation.

From the analysis of the TCGA data, we identified 472 DEMs with statistical significance, composed of 201 up-regulated miRNAs and 271 down-regulated miRNAs (Figure 1A). In addition, the analysis of the GSE24514 dataset identified 565 DEGs with statistical significance, which included 266 up-regulated genes and 299 down-regulated genes (Figure 1B).

### Cox Proportional Hazards Regression Model of DEMs

**FIGURE 2:**
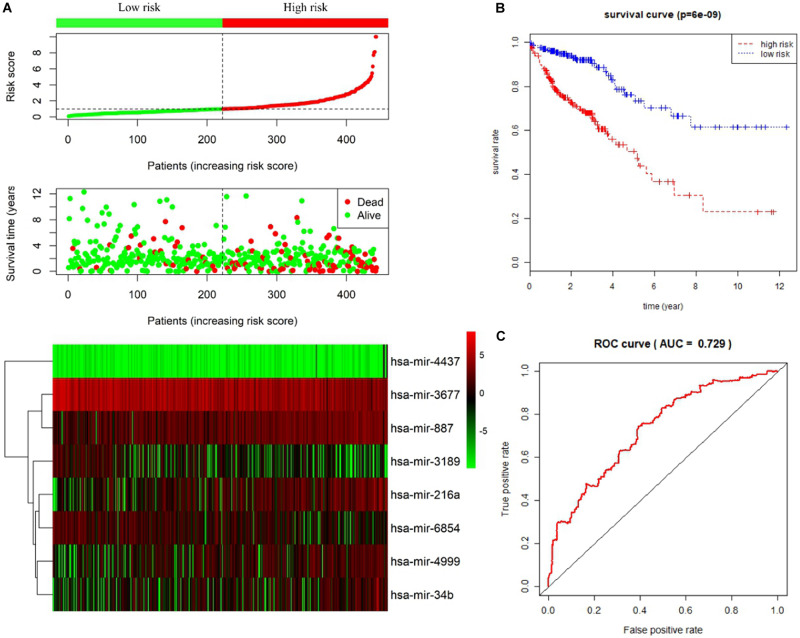
Prognostic risk score model analysis of eight prognostic miRNAs in colon cancer patients. **(A)** From top to bottom are the risk score distribution, patients’ survival status distribution, and the heatmap of eight miRNA expression profiles ranked by risk score. **(B)** Kaplan–Meier curves for high-risk and low-risk groups. **(C)** The ROC curves for predicting survival in colon cancer patients by the risk score.

Univariate and multivariate Cox proportional hazards regression analyses identified 12 miRNAs associated with survival in colon cancer patients (*P* < 0.01; Table 1) and a further 8 prognostic miRNAs (hsa-mir-6854, hsa-mir-4437, hsa-mir-216a, hsa-mir-3677, hsa-mir-887, hsa-mir-4999, hsa-mir-34b, and hsa-mir-3189), respectively (Table 2). Among prognostic miRNAs, hsa-mir-3677, hsa-mir-216a, hsa-mir-4437, and hsa-mir-6854 were also independent prognostic miRNAs (*P* < 0.05). The risk score was calculated as follows: miRNA risk score = (−0.4034 × hsa-mir-6854) + (1.6106 × hsa-mir-4437) + (0.2508 × hsa-mir-216a) + (−0.2327 × hsa-mir-3677) + (0.2306 × hsa-mir-887) + (0.2045 × hsa-mir-4999) + (0.161 × hsa-mir-34b) + (−0.2008 × hsa-mir-3189). Figure 2A presents the detailed information of the risk score. Kaplan–Meier survival analysis showed that the 5-year survival rate was 50.5% in the high-risk group and 76.3% in the low-risk group (Figure 2B). The area under the ROC curve was 0.729, demonstrating that the model could effectively predict the prognosis of colon cancer patients (Figure 2C).

**TABLE 1:** Univariate Cox regression analysis of the 12 miRNAs associated with survival in colon cancer patients.

miRNA	HR	*z*	*P-*value
hsa-mir-887	1.488449	3.418183	0.000630
hsa-mir-3677	0.729468	–3.29453	0.000986
hsa-mir-216a	1.349487	3.274952	0.001057
hsa-mir-149	1.333374	3.184117	0.001452
hsa-mir-4437	4.482079	3.068887	0.002149
hsa-mir-4999	1.390901	3.047926	0.002304
hsa-mir-1271	1.351069	2.990206	0.002788
hsa-mir-3189	0.685402	–2.91866	0.003515
hsa-mir-187	1.201949	2.841883	0.004485
hsa-mir-6854	0.726455	–2.81219	0.004921
hsa-mir-34b	1.297501	2.781959	0.005403
hsa-mir-130a	1.380213	2.744909	0.006053

*HR, hazard ratio.*

**TABLE 2:** Multivariate Cox regression analysis of the 8-miRNA signature associated with survival in colon cancer patients.

miRNA	Coefficient	HR	SE	*P-*value
hsa-mir-887	0.2306	1.2594	0.1194	0.05338
hsa-mir-3677	–0.2327	0.7924	0.1047	0.02619
hsa-mir-216a	0.2508	1.2851	0.0938	0.00750
hsa-mir-4437	1.6106	5.0059	0.4972	0.00120
hsa-mir-4999	0.2045	1.2269	0.1149	0.07519
hsa-mir-3189	–0.2008	0.8181	0.1406	0.15327
hsa-mir-6854	–0.4034	0.6681	0.1183	0.00065
hsa-mir-34b	0.1610	1.1747	0.1044	0.12306

*HR, hazard ratio; SE, standard error of coefficient.*

### Target Genes Prediction for Prognostic miRNAs

**FIGURE 3:**
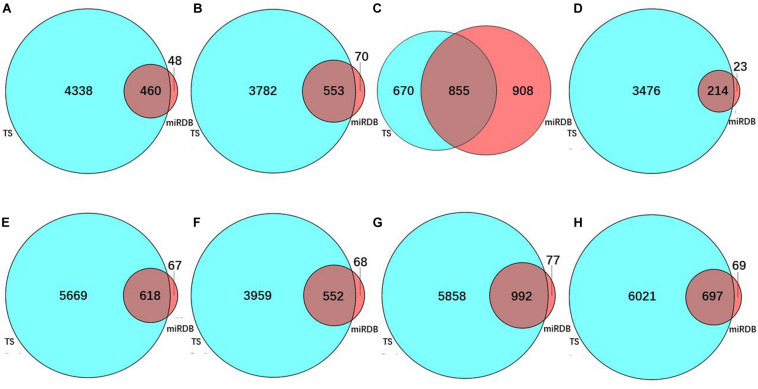
The number of predicted target genes of eight prognostic miRNAs. Target gene number predicted for **(A)** hsa-mir-6854, **(B)** hsa-mir-4437, **(C)** hsa-mir-216a, **(D)** hsa-mir-3677, **(E)** hsa-mir-887, **(F)** hsa-mir-4999, **(G)** hsa-mir-34b, and **(H)** hsa-mir-3189. In these sub-figures, blue represents the predicted results of TargetScan, and red represents the predicted results of miRDB.

To predict the target genes of the eight prognostic miRNAs, we used two independent online analytical tools (TargetScan and miRDB). Figures 3A–H shows that the intersections between the predicted results from the two servers provided 460, 553, 855, 214, 618, 552, 992, and 697 overlapping target genes of hsa-mir-6854, hsa-mir-4437, hsa-mir-216a, hsa-mir-3677, hsa-mir-887, hsa-mir-4999, hsa-mir-34b, and hsa-mir-3189, separately. Overlapping target genes of eight prognostic miRNAs and 565 DEGs from differential expression analysis of colon cancer intersected to obtain 9, 11, 14, 17, 18, 11, 30, and 19 consensus genes, respectively, for these miRNAs, with a total of 112 consensus genes (Table 3).

**TABLE 3:** One hundred and twelve consensus genes shared by the target genes of 8 prognostic miRNAs and DEGs from differential expression analysis of colon cancer.

miRNA	Consensus genes
hsa-mir-887	*SLC36A1*, *C7*, *HNRNPL*, ***MCM6***, *HSPB6*, *SVIL*, *PPP1R12B*, ***TOP2A***, *SLC17A4*, *MARCKSL1*, *ATP1A2*, *CXCL9*, *METTL7A*, *SLC25A32*, *VSNL1*, *VCL*, *FOXF1*, *CLIP3*
hsa-mir-3677	*SORBS1*, *ARNTL2*, ***KIF15***, *RAB15*, *AKAP12*, *SSR3*, *PJA2*, *CXCL12*, *CA12*
hsa-mir-216a	*CA7*, *HSPD1*, ***NEK2***, *HMGB3*, *NPTX1*, *TXNIP*, *ZCCHC24*, *CA12*, *HOXC6*, *MAN2A1*, *FOSB*
hsa-mir-4437	*CCNB1IP1*, *SPIB*, ***UBE2C***, *SULT1A1*, *CDHR5*, *HHLA2*, *BACE2*, *TRANK1*, *PTPRH*, *CD79A*, *NDC1*, *HSD11B2*, *LGR5*, *MLEC*
hsa-mir-4999	*IFITM1*, *SORBS1*, *SLC17A4*, *LMO3*, ***ZWILCH***, *CCND1*, *RCN1*, *XPOT*, *PDZRN4*, *LRRC19*, *CAV1*
hsa-mir-3189	*FAM57A*, *TMEM97*, ***FANCI***, *CDH3*, *SETBP1*, *ITIH5*, *MAB21L2*, *VIPR1*, *RETSAT*, *GOLT1B*, *MEIS1*, *NPTX1*, *JAM3*, *TXNIP*, *ZCCHC24*, *SLC4A4*, *A1CF*, *NR3C2*, *DUSP1*
hsa-mir-6854	*STMN2*, *SYNM*, *SORBS1*, ***DTL***, ***RACGAP1***, *PLN*, *C1orf115*, ***MKI67***, *GPD1L*, ***HMMR***, *SLC25A32*, *FNBP1*, *PRKACB*, *CAV1*, *TDP2*, *CXCL14*, *DCN*
hsa-mir-34b	*STMN2*, ***NCAPG2***, ***RRM2***, ***CEP55***, *CA1*, *ENC1*, *CXCL1*, *SLC17A4*, *BCAS1*, *PBX1*, *FAM47E-STBD1*, *CCDC59*, *MEST*, *MYC*, *PUS1*, *CCND1*, *SPP1*, *SATB1*, *NDC1*, *AHCYL2*, *KRT20*, *PALLD*, *MLEC*, *SSR3*, *PJA2*, *PAPSS2*, *TGFBI*, *CAV1*, *PDZRN3*, *CLDN8*

*Bold represents the critical genes.*

### Functional Enrichment Analysis of Consensus Genes

**FIGURE 4:**
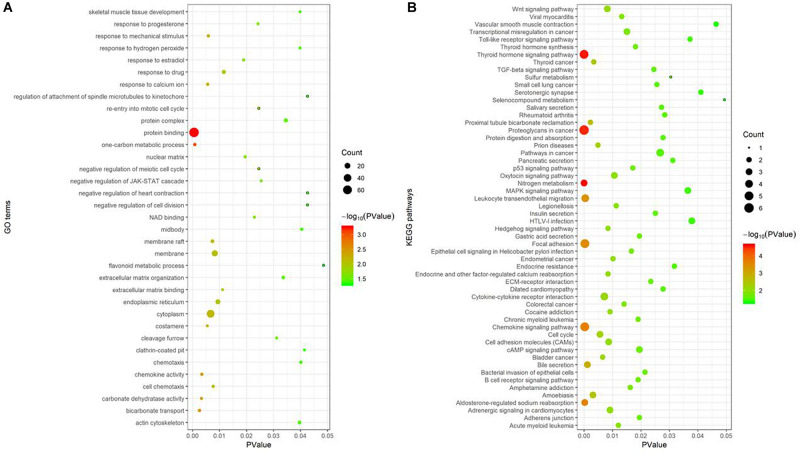
Functional enrichment analysis of 112 consensus genes. **(A)** GO enrichment analysis; **(B)** KEGG pathway enrichment analysis. In these two sub-figures, the *x*-axis represents the *P*-value, and the *y*-axis represents the different GO terms and the KEGG pathways, respectively. The size of the bubbles grows as the number of involved genes increases.

Gene Ontology enrichment analysis, performed using the DAVID database, showed 35 GO terms noticeably enriched with these 112 consensus genes included protein binding, one-carbon metabolic process, bicarbonate transport, cytoplasm, and membrane, among others (Figure 4A). The GO term “protein binding function” had the smallest *P*-value (*P* = 5.52e-04) and was enriched with the largest number of consensus genes, with a total of 72, indicating the strongest correlation between them. The KEGG pathway enrichment analysis of these consensus genes, performed using the KOBAS database, revealed 56 pathways were noticeably enriched, including nitrogen metabolism, the thyroid hormone signaling pathway, proteoglycans in cancer, chemokine signaling pathways, and focal adhesion, among others (Figure 4B). Of these pathways, nitrogen metabolism had the smallest *P*-value (*P* = 2.38e-05) and was associated with three consensus genes. Proteoglycans in cancer had the largest number of genes involved, and a *P*-value of 3.07e-05.

### Construction and Analysis of PPI Networks for Consensus Genes

**FIGURE 5:**
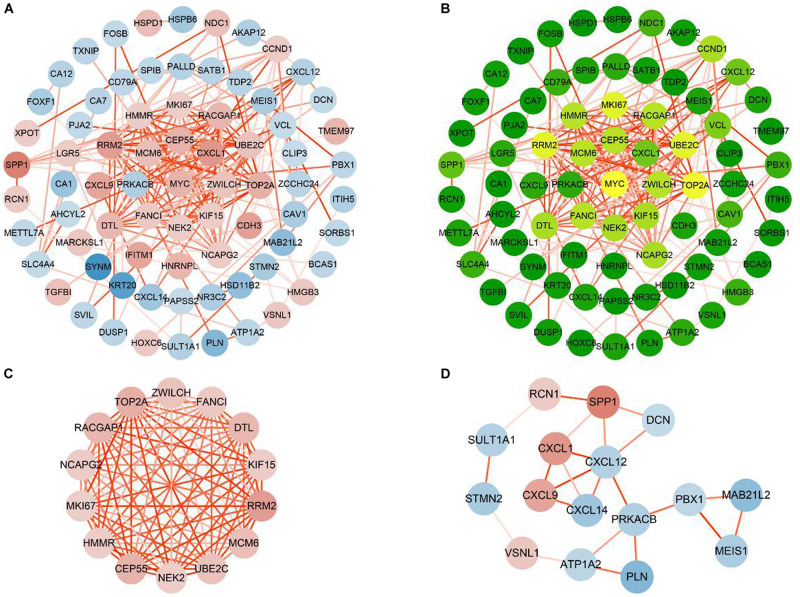
Construction and analysis PPI networks of consensus genes. **(A)** PPI network of 75 consensus genes. Red nodes represent up-regulated genes, and blue nodes represent down-regulated genes. The color of the node deepens as the value of |log2FC| increases. The color of the line connecting the circles deepens as the confidence scores increase. **(B)** Degree values of 75 consensus genes were obtained by CentiScaPe. As the degree values increase, the color of the node changes from green to yellow. **(C)** Module 1 (MCODE score = 13.8466). **(D)** Module 2 (MCODE score = 3.067).

To study their PPIs, we entered all the 112 consensus genes into the STRING database to construct the PPI network. Next, for visualization, we imported the genes with confidence scores above 0.4 into Cytoscape. The constructed network was an undirected graph. Each node in the network represented a gene, and the connections between the nodes symbolized the interactions between the proteins encoded by the corresponding genes (Kohler et al., 2008). The network contained 75 nodes and 198 interactions (Figure 5A). According to a criterion larger than twice the average (average = 5.28), we identified 16 candidate hub genes: *CCND1*, *CEP55*, *DTL*, *FANCI*, *HMMR*, *KIF15*, *MCM6*, *MKI67*, *MYC*, *NCAPG2*, *NEK2*, *RACGAP1*, *RRM2*, *TOP2A*, *UBE2C*, and *ZWILCH* (Figure 5B). The module analysis of the PPI network, performed using the MCODE plugin, revealed two functional modules (Figures 5C,D). Except for *CCND1* and *MYC*, the remaining 14 of the 16 candidate hub genes appeared in module 1, indicating that these 14 genes may play important biological functions in the PPI network, and thus, were defined as the critical genes of the network.

### Weighted Gene Co-expression Network Analysis

**FIGURE 6:**
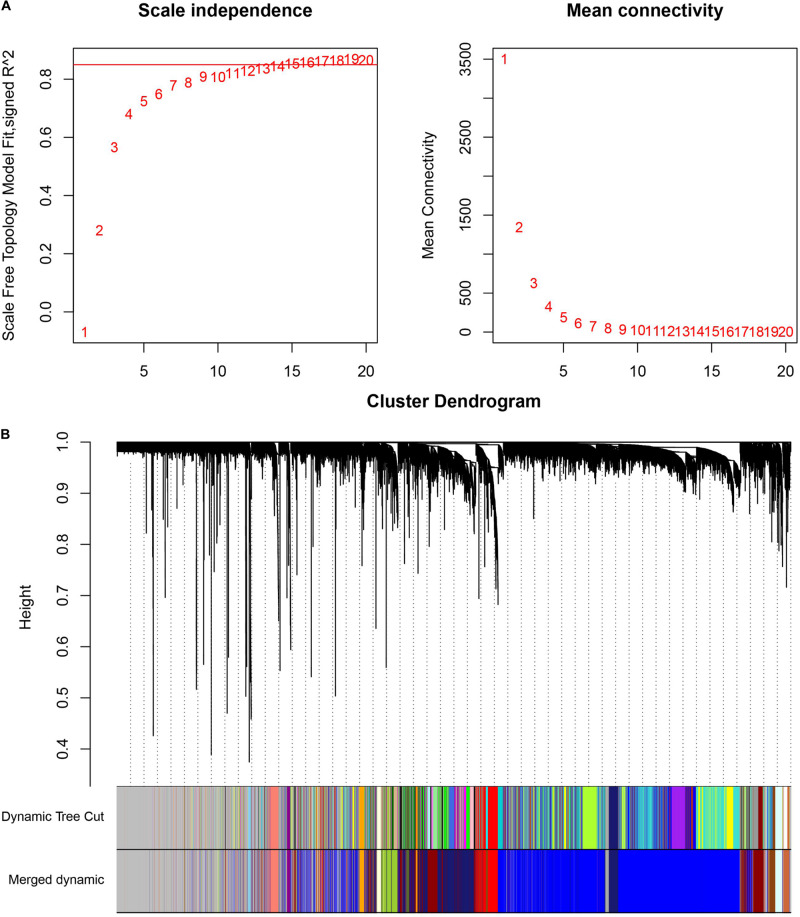
Weighted co-expression gene network analysis. **(A)** Determination of the soft threshold in the WGCNA algorithm. When the soft thresholding power was 15, the gene distribution conformed to the scale-free network. **(B)** The cluster dendrogram of all the genes in GSE24514. Each leaf represents a separate gene, and each branch represents a co-expression gene module.

**FIGURE 7:**
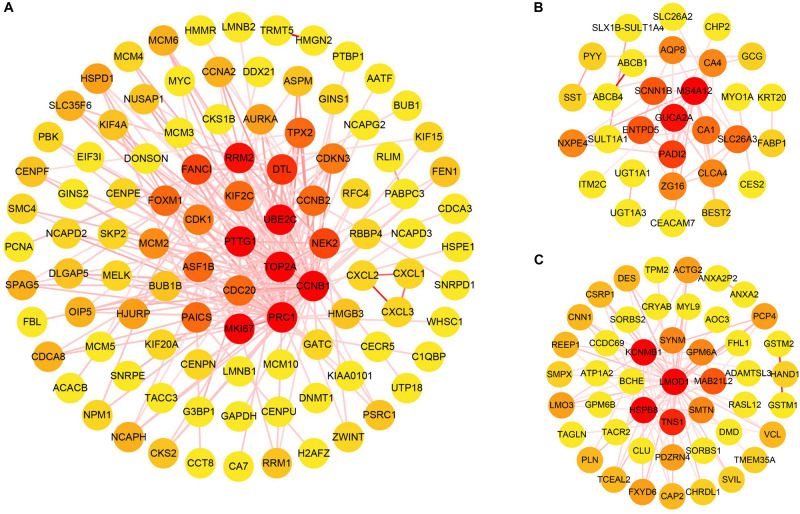
The visualization of co-expression gene modules. **(A)** Midnight blue module. **(B)** Yellow-green module. **(C)** Red module. The color of the line connecting the circles deepens as the topological overlap measures increases. The color of the node changes from yellow to red as the degree values increases.

The cluster analysis in WGCNA showed no abnormal value in the 49 GSE24514 samples. According to the independence and average connectivity of networks with different power values (power values ranging from 1 to 20), the soft threshold was determined to be 15 (Figure 6A). Ultimately, there were 17 modules of different colors generated. The co-expression degree of genes in the same module was high, and the co-expression degree of genes from different modules was low (Figure 6B). Among them, the midnight blue, red, and yellow-green modules contained the most DEGs, which were 200, 103, and 126, respectively. We constructed three weighted gene co-expression networks using edges with topological overlap measures greater than 0.30 in these modules. Ultimately, 19 core genes, which were all DEGs, were identified, according to a degree value criterion of greater than twice the average of the degree. These DEGs were *CCNB1*, *DTL*, *ENTPD5*, *FANCI*, *GUCA2A*, *HSPB8*, *KCNMB1*, *LMOD1*, *MKI67*, *MS4A12*, *NEK2*, *PADI2, PRC1*, *PTTG1*, *RRM2*, *SCNN1B*, *TNS1*, *TOP2A*, and *UBE2C* (Figures 7A–C).

## Discussion

MicroRNAs play important roles in cell differentiation, biological development, and the occurrence and progression of diseases, attracting increasing attention from researchers. Despite progress in understanding the role of miRNAs in the regulation of tumor growth and evolution, miRNAs are easily affected by a variety of factors during their activity in cancer, and they have the specificity of spatiotemporal expression in different types of tumors or different stages of the same tumors. Therefore, the specific relationships between miRNAs and tumors remain unclear and need to be further clarified.

In this study, we identified DEMs and DEGs of colon cancer from the TCGA and GEO databases, respectively. Then, we derived a prognostic model using Cox proportional hazards regression analysis based on eight miRNAs, namely hsa-miR-6854, hsa-mir-4437, hsa-mir-216a, hsa-mir-3677, hsa-mir-887, hsa-mir-4999, hsa-mir-34b, and hsa-mir-3189. We further obtained a total of 112 consensus genes from the intersection of DEGs with the target genes predicted for these eight miRNAs using TargetScan and miRDB tools. Subsequent GO and KEGG pathway enrichment analyses revealed that these consensus genes were mainly involved in protein binding, one-carbon metabolic process, nitrogen metabolism, proteoglycans in cancer, and chemokine signaling pathways. Finally, we used the STRING database to construct the PPI network of the 112 consensus genes. With the two Cytoscape plugins CentiScaPe and MCODE, 14 critical genes were recognized (*CEP55*, *DTL*, *FANCI*, *HMMR*, *KIF15*, *MCM6*, *MKI67*, *NCAPG2*, *NEK2*, *RACGAP1*, *RRM2*, *TOP2A*, *UBE2C*, and *ZWILCH*).

Among eight prognostic miRNAs in colon cancer, the expression of hsa-mir-6854, hsa-mir-216a, hsa-mir-3677, hsa-mir-4999, hsa-mir-34b, and hsa-mir-3189 was up-regulated, and that of hsa-mir-4437 and hsa-mir-887 was down-regulated. Among these eight miRNAs, hsa-mir-216a and hsa-mir-34b have been validated in experiments previously, proving they have crucial roles in colon cancer. Wang et al. (2018) showed that the up-regulation of miR-216a-3p inhibited the expression of its target genes *ALOX5* and *COX-2* in colon cancer cells, consequently enhancing the proliferation of colon cancer cells. Hiyoshi et al. (2015) used quantitative RT-PCR to detect overexpression of miR-34b in colon cancer tissues and confirmed that it was associated with poor prognosis in patients.

For the other six prognostic miRNAs, including hsa-mir-6854, hsa-mir-4437, hsa-mir-3677, hsa-mir-887, hsa-mir-4999, and hsa-mir-3189, although their roles have not yet been shown in colon cancer, some experimental studies demonstrated that the expression change of hsa-mir-887 and hsa-mir-3189 played crucial roles in other cancer cells. Jiang et al. (2016) illustrated that miR-887-5p was overexpressed in the serum of patients with endometrial cancer and might be a potential biomarker for endometrial cancer. Jones et al. (2015) demonstrated that overexpression of miR-3189-3p up-regulated p53 and many p53 target genes, which could effectively induce apoptosis and inhibit cell proliferation in colorectal cancer (CRC). In glioblastoma and gastric cancer, overexpressed miR-3189 could markedly inhibit cell proliferation and migration (Jeansonne et al., 2015; Bian et al., 2018). These studies showed miR-3189 as a tumor suppressor. The above results illustrated that the expression of miR-887 and miR-3189 in these cancers was contrary to ours. MiRNA expression may differ among cancer types, so the expression and specific mechanism of miR-887 and miR-3189 in colon cancer need to be further clarified experimentally.

He et al. (2018) showed that hsa-mir-4437 could directly act on C-X-C motif chemokine receptor 2 (CXCR2), which can increase tumor inflammation and angiogenesis. Saintigny et al. (2013) indicated that in lung adenocarcinoma, the overexpression of CXCR2 caused invasion, metastasis, and poor prognosis of tumor patients. Wu et al. (2015) also showed enhanced expression of CXCR2 in colon cancer tissues, particularly in advanced-stage tumor cells or tumor cells with lymph node metastasis, indicating the potential to use the expression level of CXCR2 for evaluating tumor growth and invasion in CRC. Our results showed that hsa-mir-4437 was an independent prognostic factor for colon cancer, and CXCR2 was found from the target prediction by both TargetScan and miRDB. Therefore, hsa-mir-4437 may affect the proliferation and apoptosis of colon cancer cells by targeting CXCR2.

According to our prediction results, all 14 critical genes of colon cancer we identified from the PPI network were up-regulated in colon cancer cells. Among these 14 genes, abnormal overexpression of *CEP55*, *DTL*, *HMMR*, *MCM6*, *MKI67*, *NEK2*, *RACGAP1*, *RRM2*, *TOP2A*, and *UBE2C* have previously been reported in colon cancer (Table 4).

**TABLE 4:** Fourteen critical genes reported in cancer from previous studies.

Gene	*CEP55*	***DTL***	***FANCI***	*HMMR*	*KIF5*	*MCM6*	***MKI67***
Feature	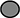	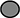	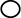	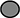	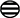	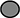	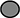
Gene	*NCAPG2*	***NEK2***	*RACGAP1*	***RRM2***	***TOP2A***	***UBE2C***	*ZWILCH*
Feature	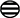	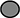	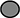	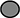	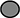	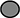	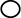

*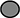 Gene experimentally up-regulated in colon cancer, and the expression was consistent with our calculation result of colon cancer. 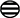 Gene experimentally up-regulated in other cancers, and the expression was consistent with our calculation result of colon cancer. 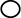 Gene not yet experimentally verified in colon cancer or other cancers. Bold represents the genes that are both critical genes and WGCNA core genes.*

Denticleless E3 ubiquitin–protein ligase homolog (DTL) complex is a nuclear protein that targets centrosomes in mitosis, with an important role in DNA synthesis, cell cycle regulation, cytokinesis, proliferation, and differentiation (Pan et al., 2006). Baraniskin et al. (2012) demonstrated that miR-30a-5p could produce a tumor suppressor effect by repressing the overexpression of DTL in colon cancer. Karaayvaz et al. (2011) showed miR-215 achieved a similar outcome. The Rac GTPase activating protein 1 (RACGAP1) is a member of the GTPase-active protein family, with a regulatory role in cell division, cell growth differentiation, and tumor metastasis and proliferation (Milde-Langosch et al., 2013; Yeh et al., 2016). According to Yeh et al. (2016), patients with high expression of cytoplasmic RACGAP1 in CRC had a favorable prognosis, whereas those with high expression of nuclear RACAGAP1 had a poor prognosis. Imaoka et al. (2015) demonstrated that RACGAP1 expression was dramatically high in CRC with advanced tumor stage, vessel invasion, and lymph node and distant metastasis, causing poor overall survival. The *marker of proliferation Ki-67* (*MKI67*) is a nucleoprotein gene involved in cell proliferation and expressed at all stages of the cell cycle (Yang et al., 2017). Lin et al. (2008) detected high expression of MKI67 in CRC based on immunohistochemistry. Zeng et al. (2019) showed that in CRC, the knockdown of oncogenic gene *small nuclear ribonucleoprotein polypeptide A* (*SNRPA1*) caused the down-regulation of its other downstream genes, including *MK167*, inhibiting the proliferation of CRC cells. The hyaluronan-mediated motility receptor (HMMR), also known as RHAMM, plays a key role in the occurrence and development of tumors by mediating the migration of hyaluronan to tumor cells and is closely related to cell proliferation, migration, signal transduction, adhesion, and metastasis (Hatano et al., 2011). Koelzer et al. (2015) indicated that HMMR was overexpressed in tumor-budding cells of CRC and associated with advanced tumor grade, invasion, metastasis, and poor prognosis. HMMR is also a biomarker for poor prognosis in several cancers, including those of the colon, stomach, lung, and breast (Chen et al., 2018).

The ubiquitin-conjugating enzyme E2C (UBE2C) is the central component of the ubiquitin–proteasome system (UPS), an ATP-dependent protein degradation pathway in the cytoplasm and nucleus (Rousseau and Bertolotti, 2018). By immunohistochemical analysis, Fujita et al. (2009) confirmed that the UBE2C content was higher in colon cancer tissues than in normal colon epithelium, and overexpressed UBE2C could change the cell cycle and promote tumor proliferation. Okamoto et al. (2003) noted that UBE2C was highly expressed in a variety of tumors, including CRC, causing cell growth promotion and malignant transformation. *NIMA-related kinase 2* (*NEK2*) encodes a serine/threonine protein kinase involved in the centrosome cell cycle and mitosis regulation. The expression of *NEK2* is closely associated with the prognosis and pathological features of cancer, including colon cancer (Ren et al., 2018). Takahashi et al. (2014) found that the high expression of *NEK2* in CRC was associated with advanced tumor stage, invasion, dissemination, and poor prognosis, but that mir-128 could repress *NEK2* expression, and inhibited cell proliferation. As a member of the MCM family, *mini-chromosome maintenance complex component 6* (*MCM6*) is highly expressed in human malignant cells. The encoded product of *MCM6* is a key protein for DNA replication and is involved in the regulation of the cell cycle (Lei, 2005). *MCM6* is highly expressed in colon cancer tissues (Hendricks et al., 2019). Huang et al. (2018) showed that the suppression of *MCM6* in colon cancer cells could inhibit the foci-forming and chromatin localization of RAD51 recombinase (RAD51), a protein essential for DNA damage recovery. DNA topoisomerase II alpha (TOP2A) is a key enzyme that controls the topological state of DNA and is involved in processes, such as chromosome condensation, chromatid separation, and gene expression (Tsavaris et al., 2009). Zhang R. et al. (2018) detected up-regulated TOP2A in colon cancer tissues compared with adjacent non-cancerous tissues and found that down-regulated TOP2A in colon cancer cells could dramatically inhibit proliferation and invasion of colon cancer cells. The ribonucleotide reductase regulatory subunit M2 (RRM2) plays a vital role in DNA synthesis and repair, as well as many key cellular processes, such as cell proliferation, invasion, migration, senescence, and tumorigenesis (Nordlund and Reichard, 2006). In colon cancer, the increased expression of RRM2 can noticeably enhance the invasive ability of cancer cells (Liu et al., 2007). Liu et al. (2013) proved that increasing the expression of RRM2 in colon cancer cells substantially enhanced cell migration and invasion ability, which indicated that RRM2 was an independent prognostic biomarker for colon cancer and could predict the low survival rate of colon cancer patients. The centrosomal protein 55 (CEP55) is involved not only in the process of cytokinesis but also in the invasion, metastasis, and prognosis of many malignancies (Jeffery et al., 2016). Bioinformatics analysis performed by Hauptman et al. (2019) indicated that CEP55 was overexpressed in CRC and could be used as a potential biomarker in colon cancer tissues, as validated in clinical samples. Similarly, Sakai et al. (2006) reported that inhibiting the expression of CEP55 caused a marked reduction in the growth rate of colon cancer cells. These experiment results on the expression of the above 10 genes in colon cancer are consistent with our calculation results, which further verified the reliability of our computational analysis.

As mentioned above, 4 of the 14 key genes recognized in this study as playing major roles in colon cancer (*FANCI*, *KIF15*, *NCAPG2*, and *ZWILCH*) have not been experimentally shown to be up-regulated in colon cancer. However, abnormal overexpression of *KIF15* and *NCAPG2* has been detected in many other types of cancer. Kinesin family member 15 (KIF15) is involved in important biological processes, including mitosis, cell signaling pathways, gene translation, and protein trafficking (Penna et al., 2017). According to Sheng et al. (2019), the up-regulation of *KIF15* in breast cancer led to poor overall survival, indicating that *KIF15* could serve as a potential therapeutic target for triple-negative breast cancer. In a study by Wang et al. (2017), overexpression of *KIF15* in pancreatic cancer promoted the expression of p-MEK and p-ERK, inducing activation of the MEK–ERK signaling pathway and causing G_1_/S phase transition and cancer growth. The non-SMC condensin II complex subunit G2 (NCAPG2) is a component of the condensin II complex that interacts with Polo-like kinase 1 (PLK1) during the anterior-to-metaphase transition of mitosis, thereby regulating correct chromosome segregation (Kim et al., 2014). The up-regulation of *NCAPG2* in non-small cell lung cancer (NSCLC) cells caused a short survival time, whereas suppressing *NCAPG2* expression led to proliferation inhibition and G_2_/S cycle arrest (Zhan et al., 2017). Zhan et al. (2017) concluded that *NCAPG2* expression was closely related to the progression of NSCLC and could act as a prognostic factor. In liver cancer, Meng et al. (2019) determined that highly expressed *NCAPG2* promoted tumor cell proliferation, migration, and invasion, mediated by activation of the STAT3 and NF-κB pathways. Such findings confirmed *NCAPG2* as both an oncogene of liver cancer and a biomarker predicting poor patient prognosis. In summary, *KIF15* and *NCAPG2* might be involved in the development and progression of colon cancer, and they serve as prognostic markers or therapeutic targets for colon cancer.

Zwilch kinetochore protein (ZWILCH) is an important component of the Rod–Zw10–Zwilch complex and is crucial for maintaining the normal function of mitotic checkpoints (Kops et al., 2005). The abnormal function of mitotic checkpoints is associated with the appearance of chromosomal instability, a consensus sign of many human malignancies. According to Shih et al. (2001), chromosomal instability occurs in the early stages of colon cancer, resulting in genomic instability that might promote tumor development. *Fanconi anemia* (*FA*) *complementation group I* (*FANCI*) is a gene belonging to the FA–breast cancer pathway, and the mono-ubiquitination of the FANCI–FANCD2 protein complex is the key to the normal function of the FA pathway (Smogorzewska et al., 2007). A dysfunctional FA pathway reduces the ability of DNA repair, causing genomic instability, which increases the incidence of tumor development (Deans and West, 2011). *FANCI* is one of the most pathogenic mutated genes in CRC (Zhunussova et al., 2019). This gene is a negative regulator of the consensus oncogene *Akt* (Zhang et al., 2016). Although no current study concerns the direct correlation between these two genes and cancer, both genes can act as components of cancer progression pathways and play certain roles in the formation of cancer, yet the specific mechanism remains unclear. Our results identified *FANCI* and *ZWILCH* as critical target genes of colon cancer, suggesting that they might provide a potential pathway for the treatment and intervention of colon cancer.

In this study, we also performed WGCNA to mine the core genes via the analysis of gene expression patterns of multiple samples in GSE24514 and constructed 17 co-expression modules from 13,640 genes of these transcriptome data. Among them, three modules (midnight blue, red, and yellow-green) containing the most DEGs were the key functional ones significantly related to colon cancer. These modules comprised 19 core genes (*CCNB1*, *DTL*, *ENTPD5*, *FANCI*, *GUCA2A*, *HSPB8*, *KCNMB1*, *LMOD1*, *MKI67*, *MS4A12*, *NEK2*, *PADI2*, *PRC1*, *PTTG1*, *RRM2*, *SCNN1B*, *TNS1*, *TOP2A*, and *UBE2C*). 7 genes, including *DTL*, *FANCI*, *MKI67*, *NEK2*, *RRM2*, *TOP2A*, and *UBE2C*, appeared in the above-discussed 14 critical genes from the consensus genes (Table 4), which, at least partially verified the reliability of the main results of this work. In addition, although the remaining 12 core genes from WGCNA were not target genes for our derived prognostic miRNAs, they were DEGs of colon cancer, and their relationship with colon cancer deserves further study.

Bioinformatics is indispensable for mining data related to colon cancer. In 2018, Wei et al. (2018) constructed a 10-miRNA prognostic model composed of hsa-mir-891a, **hsa-mir-6854**, **hsa-mir-216a**, hsa-mir-378d-1, hsa-mir-92a-1, hsa-mir-4709, hsa-mir-92a-2, hsa-mir-210, hsa-mir-940, and **hsa-mir-887**, by analyzing the genome-wide miRNA sequencing dataset and corresponding clinical information of 425 colon adenocarcinoma patients from TCGA. In the same year, Zhang H. et al. (2018) identified DEMs between 457 colon cancer tissues and 8 normal tissues from TCGA. Subsequent Cox proportional hazards regression analysis provided a prognostic model of six miRNAs, including hsa-mir-149, **hsa-mir-3189**, **hsa-mir-3677**, hsa-mir-3917, **hsa-mir-4999**, and **hsa-mir-6854**. Thus, six of eight prognostic factors (**hsa-mir-6854**, hsa-mir-4437, **hsa-mir-216a**, **hsa-mir-3677**, **hsa-mir-887**, **hsa-mir-4999**, hsa-mir-34b, and **hsa-mir-3189**) we calculated were consistent with their results. It is worth noting that although the expressions of miR-887 and miR-3189 in other cancers were experimentally different from our predicted ones, the above two studies provided the same analysis results as ours showed in colon cancer, namely down-regulated miR-887 and up-regulated miR-3189. However, different from these two previous works, our study further explored the potential critical target genes of the prognostic miRNAs by using combination methods, including target prediction calculation, differential expression screening, intersection analysis, and PPI network construction and visualization.

As shown in Table 3, 14 targeting relationships are available between 8 prognostic miRNAs and 14 critical genes. Specifically, *DTL*, *HMMR*, *MKI67*, and *RACGAP1* were predicted as the target genes of hsa-mir-6854. *FANCI*, *KIF15*, *NEK2*, *UBE2C*, and *ZWILCH* were the target genes of hsa-mir-3189, hsa-mir-3677, hsa-mir-216a, hsa-mir-4437, and hsa-mir-4999, respectively. *MCM6* and *TOP2A* were the target genes of hsa-mir-887, and *CEP55*, *NCAPG2*, and *RRM2* were the target genes of hsa-mir-34b (Table 3). To date, there have been no experiments to confirm any of the above targeting relationships. However, the targeting relationships of *KIG15* with has-mir-3677 (Zorniak et al., 2018), and *CEP55* with has-mir-34b (Liang, 2008) were also predicted in the functional analysis of miRNA in patients with gastric antral vascular ectasia and expression meta-analysis of lung cancer miRNA targets, respectively. As discussed above, experiments have shown that these miRNAs and genes are mostly, directly or indirectly, related to colon cancer. Therefore, we speculate the existence of these targeting relationships for further study, which might clarify the mechanisms of colon cancer and provide novel methods for future exploration of prevention and treatment.

## Conclusion

In summary, we used bioinformatics methods to construct a prognostic model of colon cancer patients with eight prognostic miRNAs, including hsa-mir-6854, hsa-mir-4437, hsa-mir-216a, hsa-mir-3677, hsa-mir-887, hsa-mir-4999, hsa-mir-34b, and hsa-mir-3189. Fourteen potential critical target genes of these independent prognostic biomarkers were identified in the PPI network. These genes were *CEP55*, *DTL*, *FANCI*, *HMMR*, *KIF15*, *MCM6*, *MKI67*, *NCAPG2*, *NEK2*, *RACGAP1*, *RRM2*, *TOP2A*, *UBE2C*, and *ZWILCH*. One miRNA (hsa-mir-4437) and four genes (*FANCI*, *KIF15*, *NCAPG2*, and *ZWILCH*) have not yet been confirmed to be associated with colon cancer in previous experiments and calculations. In addition, the targeting relationship between the 8 prognostic miRNAs and the 14 critical genes deserves further study. Furthermore, 12 core genes obtained from WGCNA are also worthy of future research. Our results indicate that these prognostic miRNAs and their target genes could have valuable potential for prognosis and targeted therapy of colon cancer, and thereby could provide new guidance for the diagnosis and treatment of colon cancer in the future.

## Data Availability Statement

Publicly available datasets were analyzed in this study. These data can be found here: https://portal.gdc.cancer.gov/ and https://www.ncbi.nlm.nih.gov/geo/query/acc.cgi?acc=GSE24514.

## Author Contributions

WC, CG, and ZH contributed to the design and conception of the study. WC, CG, YL, YW, and XH did information retrieval and analysis. WC and CG wrote the manuscript. WC, YL, YW, and XH created tables and figures. ZH guided manuscript writing, revised the manuscript and provided financial support. All authors contributed to manuscript revision, read and approved the submitted version.

## Conflict of Interest

The authors declare that the research was conducted in the absence of any commercial or financial relationships that could be construed as a potential conflict of interest.

## Abbreviations

CBL: Cbl proto-oncogene
CCNB1: cyclin B1
CEP55: centrosomal protein 55
CIN: chromosomal instability
COAD: colon adenocarcinoma
CXCR2: C-X -C motif chemokine receptor 2
DAVID, Database for Annotation: Visualization and Integrated Discovery
DEGs: differentially expressed genes
DEMs: differentially expressed miRNAs
DTL: denticleless E3 ubiquitin protein ligase homolog
ENTPD5: ectonucleoside triphosphate diphosphohydrolase 5
FANCI: FA complementation group I
GEO: Gene Expression Omnibus Database
GO: Gene Ontology Database
GUCA2A: guanylate cyclase activator 2A
HMMR: hyaluronan mediated motility receptor
HSPB8: heat shock protein family B (small) member 8
KCNMB1: potassium calcium-activated channel subfamily M regulatory beta subunit 1
KEGG: Kyoto Encyclopedia of Genes and Genomes Database
KIF15: kinesin family member 15
KOBAS: KEGG Orthology Based Annotation System
LMOD1: leiomodin 1
MCM6: minichromosome maintenance complex component 6
MCODE: Molecular Complex Detection
MiRNAs: microRNAs
MKI67: marker of proliferation Ki-67
MS4A12: membrane spanning 4-domains A12
NCAPG2: non-SMC condensin II complex subunit G2
NEK2: NIMA related kinase 2
NSCLC: non-small cell lung cancer
PADI2: peptidyl arginine deiminase 2
PLK1: polo-like kinase 1
PPI: protein–protein interaction
PPP2CA: protein phosphatase 2 catalytic subunit alpha
PRC1: protein regulator of cytokinesis 1
PTTG1, PTTG1 regulator of sister chromatid separation: securing
RACGAP1: Rac GTPase activating protein 1
RAD51: RAD51 recombinase
ROC: receiver operating characteristic
RRM2: ribonucleotide reductase regulatory subunit M2
SCNN1B: Sodium channel epithelial 1 subunit beta
SMAD4: SMAD family member 4
SNRPA1: small nuclear ribonucleoprotein polypeptide A
STRING: Search Tool for the Retrieval of Interacting Genes
TCGA: The Cancer Genome Atlas Database
TNBC: triple-negative breast cancer
TNS1: Tensin 1
TOP2A: DNA topoisomerase II alpha
UBE2C: ubiquitin conjugating enzyme E2 C
UPS: ubiquitin–proteasome system
WGCNA: weighted gene co-expression network analysis
ZEB1, zinc finger E-box binding homeobox 1. ZWILCH: zwilch kinetochore protein.
